# The Response of Plants and Mycorrhizal Fungi to Nutritionally-Heterogeneous Environments Are Regulated by Nutrient Types and Plant Functional Groups

**DOI:** 10.3389/fpls.2021.734641

**Published:** 2021-11-15

**Authors:** Bitao Liu, Fei Han, Kaixiong Xing, Aiping Zhang, Zed Rengel

**Affiliations:** ^1^College of Forestry, Shanxi Agricultural University, Taigu, China; ^2^Center for Forest Ecosystem Studies and Qianyanzhou Ecological Station, Key Laboratory of Ecosystem Network Observation and Modeling, Institute of Geographic Sciences and Natural Resources Research, Chinese Academy of Sciences, Beijing, China; ^3^Institute of Environment and Sustainable Development in Agriculture, Chinese Academy of Agricultural Sciences, Beijing, China; ^4^Soil Science and Plant Nutrition, UWA School of Agriculture and Environment, The University of Western Australia, Perth, WA, Australia; ^5^Institute for Adriatic Crops and Karst Reclamation, Split, Croatia

**Keywords:** nutrient patch, mycorrhizal fungi, foraging precision, herbaceous species, woody species, root diameter

## Abstract

Nutrient type and plant functional group are both important in influencing proliferation of roots or hyphae and their benefit to plant growth in nutritionally heterogeneous environments. However, the studies quantifying relative importance of roots vs. hyphae affecting the plant response to nutrient heterogeneity are lacking. Here, we used meta-analysis based on 879 observations from 66 published studies to evaluate response patterns of seven variables related to growth and morphological traits of plants and mycorrhizal fungi in nutritionally heterogeneous environments. We found that phosphorus [P] and organic fertilizer [OF] supply significantly increased shoot (+18.1 and +25.9%, respectively) and root biomass (+31.1 and +23.0%, respectively) and root foraging precision (+11.8 and +20.4%, respectively). However, there was no significant difference among functional groups of herbs (grasses, forbs, and legumes), between herbs and woody species, and between arbuscular mycorrhizal (AM) and ectomycorrhizal (ECM) tree species in the shoot, root and mycorrhizal fungi responses to nutrient heterogeneity, except for root biomass and root foraging precision among grasses, forbs, and legumes, and mycorrhizal hyphal foraging precision between AM and ECM tree species. Root diameter was uncorrelated with neither root foraging precision nor mycorrhizal hyphal foraging precision, regardless of mycorrhizal type or nutrient type. These results suggest that plant growth and foraging strategies are mainly influenced by nutrient type, among other factors including plant functional type and mycorrhizal type.

## Introduction

In soils, nutrient distribution is spatially heterogeneous (Waring et al., 2020). Root and mycorrhizal hyphae proliferation within nutrient-rich zones is a ubiquitous adaptive strategy to acquire nutrients quickly (Hodge, 2004, 2006) and promote plant growth to confer a competitive advantage to species with high root plasticity (Hodge et al., 1999; Kembel et al., 2008; Mommer et al., 2012; Li H. et al., 2019; Zhang et al., 2019). Kembel and Cahill (2005) found that capacity to respond to small nutrient patches was generally higher in roots of dicots than monocots, suggesting it was phylogenetically and taxonomically conserved. In addition, root proliferation is also generally nutrient-specific (Robinson, 1994a; Forde and Lorenzo, 2001; Liu et al., 2015), especially when plant species adopt different strategies to obtain different nutrients (Ceulemans et al., 2017; Nasto et al., 2017; Wang et al., 2021). For example, Li et al. (2014) found that localized supply of phosphate (P) significantly increased wheat root length, whereas localized supply of nitrogen (N) had only a relatively small effect. Therefore, nutrient-specific (e.g., N, P) responses of plant species may modify phylogenetic signals in root responses to nutritionally heterogeneous environments.

Previous studies found that large variation in the diameter of absorptive fine roots (e.g., first-, second-, and sometime third-order roots) across plant species can affect plant responses to nutritionally heterogeneous environments (Farley and Fitter, 1999; Eissenstat et al., 2015; Liu et al., 2015; Chen et al., 2018). The thin-rooted species are considered to have higher specific root length (Kramer-Walter et al., 2016) and root growth rate (Eissenstat et al., 2015), and even greater root plasticity or foraging precision than species with thick roots (Chen et al., 2016). Generally, the herbaceous species tend to have thinner fine-roots than woody species (Freschet et al., 2017). Given that, herbaceous species are likely to have higher root foraging precision, capture more nutrients and accrue greater growth benefits from the nutrient patches in comparison with woody species. As we known, however, greater plasticity in thin-rooted than thick-rooted species was observed among four grass and legumes crop species in localized N+P supply (Li et al., 2014); other studies found no significant correlation between growth rate and root foraging precision across plant species in natural forest systems (Eissenstat et al., 2015; Liu et al., 2015) and grasslands (Johnson and Biondini, 2001; Grime and Mackey, 2002). Based on these studies, we hypothesized that differential effects of nutrient heterogeneity on herbaceous and woody plants may be smaller than expected, especially when considering the interference of nutrient-specific plant responses. However, few studies have systematically quantified the difference in influence of heterogeneous nutrient distribution on the shoot and root growth of herbaceous and woody species (Robinson, 1994a).

Root proliferation in nutrient patches can be altered by mycorrhizal fungi. Several recent studies demonstrated there were tradeoffs between roots and mycorrhizal fungi in nutrient capturing strategies across forest, grassland and crop species with different root diameter (Eissenstat et al., 2015; Liu et al., 2015; Li et al., 2017; Wen et al., 2019) and between different nutrient additions for one species (Li L. et al., 2019; Wang et al., 2020). Plants may differentially balance allocating photosynthates to root and/or mycorrhizal hyphal proliferation in the nutrient patches. For example, hyphal proliferation of arbuscular mycorrhizal (AM) fungi in the organic matter patches can be restricted in the presence of roots (Hodge and Storer, 2015). Recent study also found that root length density of Chinese fir decreased with P addition, and there was no significant change after the addition of N, whereas hyphal length density decreased with addition of N (Li L. et al., 2019). Based on that, testable questions would be: (1) whether thin-rooted species would have higher root plasticity but lower hyphal plasticity than thick-rooted species in nutrient patches; and (2) whether such a correlation would depend on the attributes of nutrient patches.

The optimal nutrient foraging strategy (reliant on roots or mycorrhizal hyphae) may also depend on whether a species is associated with either AM or ectomycorrhizal (ECM) fungi. Ectomycorrhizal tree species generally have greater root branching intensity and thinner roots than AM tree species, but there is wide variation within both categories (Comas et al., 2014). According to the above discussion, ECM tree species should have relatively higher root plasticity than AM tree species. However, ECM tree species are considered to rely more on mycorrhizal hyphae and less on roots than AM plant species in foraging nutrients from soil layers rich in organic matter (Chen et al., 2016). This is because ECM fungi are not just the extension of roots; instead, they have a superior capacity relative to roots or AM fungi to facilitate decomposition of organic matter and acquire the nutrients released (Smith and Read, 2008; Shah et al., 2016; Liu et al., 2018). This may be why ECM trees exhibit higher hyphal foraging precision in the organic than inorganic patches (Cheng et al., 2016). Therefore, the distinction of foraging strategies between AM and ECM tree species may be complex and mainly dependent on nutrient conditions.

The previous reviews have focused on various aspects of plant responses to the nutrient patches (Robinson, 1994a; Hutchings and John, 2004; Hodge, 2004, 2006; Kembel and Cahill, 2005; Cahill and McNickle, 2011). However, some questions remain unanswered. For example, Kembel and Cahill (2005) found little evidence of root foraging precision promoting plant growth in the nutritionally heterogeneous relative to the homogeneous soil. However, their conclusion could have been confounded by variable spatial patterns and amounts of nutrient supplied [the data coming mainly from Johnson and Biondini (2001) whereby total nutrient supply was higher in the homogeneous than the heterogeneous treatment]. Importantly, a relatively low total nutrient amount in the heterogeneous treatment (Johnson and Biondini, 2001) may have enhanced root foraging precision because a high shoot nutrient demand (systemic signal) could strengthen the local response and induce root proliferation in the patch zone (Forde and Lorenzo, 2001; de Kroon et al., 2009). Moreover, root foraging precision exhibited a phylogenetically conserved pattern only in the plant species studied by Johnson and Biondini (2001), rather than in the combined species from multiple studies (Kembel and Cahill, 2005). Thus, the current reviews (Wang et al., 2021) underline large variation in responses of plants to soil nutrient heterogeneity, but significant quantitative patterns are yet to be discovered. This calls for a proper meta-analysis of numerous studies available in the literature, taking into account factors that might potentially obscure important patterns.

To quantify the response of plants to nutrient heterogeneity, we selected from the previously published studies seven key parameters related to the growth and traits of plants and mycorrhizal fungi including biomass allocation of shoots and roots, the proliferation of roots and mycorrhizal hyphae, mycorrhizal colonization and root diameter. We aimed to address the following questions: (1) whether different nutrient types can explain the variation in plant responses to heterogeneous nutrient distribution, (2) whether plant responses to heterogeneously supplied nutrients would vary among different functional groups of herbaceous plants, between herbs and woody plants, and between AM and ECM tree species, and (3) whether there would be relationships between root diameter and root or mycorrhizal hyphal foraging precision in response of plant species to heterogeneous nutrient distribution in soil.

## Materials and Methods

### Selection of Studies

To develop a comprehensive database, we searched peer-reviewed papers listed in The Science Citation Index Expanded database (dating from 1990 to 2021) with the following keywords: (*plant biomass* OR *shoot biomass* OR *root biomass* OR *root length* OR *root proliferation* OR *root plasticity* OR *mycorrhizal hyphal proliferation* OR *mycorrhizal hyphal plasticity* OR *mycorrhizal hyphal biomass* OR *mycorrhizal hyphal length* OR *mycorrhizal colonization* OR *foraging strategy* OR *foraging precision* OR *foraging behavior*) AND (*localized nutrient* OR *nutrient patch* OR *heterogeneous nutrient* OR *non-uniform nutrient* OR *nitrogen patch* OR *phosphorus patch*). We extracted papers from our search that matched the following criteria: (i) plant species included herbs (grasses, non-leguminous forbs, and legumes) and woody plants (AM and ECM tree species) grown in either greenhouse or field conditions; (ii) the same or very similar amounts of nutrients supplied in the homogenous and the heterogeneous treatments, especially when evaluating plant biomass allocation and foraging strategy in nutritionally heterogeneous soils, and (iii) the nutrients considered were N (nitrate or ammonium alone), P (inorganic phosphorus alone), NP (N and P) or OF (organic fertilizer or organic matter). With respect to the criterion (ii) above, we excluded the data from Johnson and Biondini (2001) (49 species) and Grime and Mackey (2002) (43 species) because they featured the split-pot designs with vastly different nutrient amounts between the homogeneous and the heterogeneous treatments. Additionally, we excluded the articles that only reported the total biomass of all plants in different plant functional groups, and articles that nutritional treatment only set different types of heterogeneous nutrients without homogeneous nutrient or deionized H_2_O as control. Furthermore, all selected studies were divided into two datasets: the heterogeneous nutrients in the first dataset were compared with homogeneous nutrients with the same nutrient amounts for herbaceous or woody plants in greenhouse or field; while the heterogeneous nutrients (i.e., nutrient-rich patch) in the second dataset were compared with unfertilized control for woody species in the field (details in Supplementary Appendix 1 in the Supplementary Material). Specifically, the articles for the second dataset must contain root growth (root length or biomass or foraging precision), mycorrhizal fungi growth (hyphal length or biomass or foraging precision, or mycorrhizal colonization) and root diameter. For mycorrhizal type, species were classified into AM or ECM based on the reports of each study; if not be reported, mycorrhizal type was designated according to previous reviews (Wang and Qiu, 2006; Soudzilovskaia et al., 2020). A total of 879 observations from 66 published studies with 142 plant species (90 herbaceous, 43 AM and 9 ECM woody species) were used in this meta-analysis (see Supplementary Material for details). The aim of the first dataset was to test whether and how biomass allocation of shoots and roots, as well as root foraging precision would vary among different nutrient types (N, P, NP, and OF), among functional groups of herbaceous plants (grasses, forbs, and legumes), and between herbs and woody plants. The aim of the second dataset was to test whether the foraging precision of roots and mycorrhizal hyphae would vary among different nutrient types (N, P, NP, and OF) and between AM and ECM woody plants; moreover, whether the foraging precision of roots and mycorrhizal hyphae was influenced by root diameter.

### Data Collection

From each study that met the above criteria, at least one from seven parameters listed as below was reported to describe plant responses to non-uniform nutrient supply. The seven key parameters contained the following variables: four variables related to plant growth traits (shoot biomass, root biomass, root:shoot biomass ratio, root length, or biomass proliferation for calculating root foraging precision); two variables related to mycorrhizal fungi (mycorrhizal hyphae length or biomass proliferation for calculating mycorrhizal hyphal foraging precision, and mycorrhizal colonization [i.e., the frequency of colonization]); and one variable related to root morphological trait (the diameter of absorptive fine roots including first two or three orders). Here, we adopted foraging precision of roots and mycorrhizal hyphae to assess the response of plants and mycorrhizal fungi to heterogeneous nutrient patches, because this parameter, which being phylogenetic conservative, can character plant strategy of resource acquisition (de Kroon and Mommer, 2006).

For each parameter, we extracted the mean, standard deviation (SD) and the number of replicates (*n*) for the controls (homogeneous nutrient treatment in the first dataset and unfertilized treatment in the second dataset) and the heterogeneous nutrient patches. If standard errors (SE) were reported, these were transformed according to the equation SD = SE × *n*^0.5^. Unspecified error bars in the studies were assumed to represent standard error. When necessary, data were taken from graphs using the GETDATA software (v.2.26^1^).

### Statistical Analysis

For the biomass allocation of shoots and roots, as well as the mycorrhizal colonization, effect size (*EZ*) was calculated as the natural log-transformed response ratio [ln(*RR*)] in the nutrient patch treatment compared with the control using eqn 1 (Hedges et al., 1999). For foraging precision (*FP*) of roots and mycorrhizal hyphae (i.e., the ability of root and mycorrhizal hyphal proliferation into a nutrient patch), effect size was calculated using eqn 2, consistent with most studies (Mou et al., 1997; Einsmann et al., 1999; Armas et al., 2004). As previous study has shown that ln*RR* has similar properties to *FP* (Armas et al., 2004).


(1) $$In\left(RR\right)=In\left(M_{t}/M_{c}\right)$$

(2) $$\text{FP}\left(\%\right)=100\%\times \left(M_{t}-M_{c}\right)/\left(M_{t}+M_{c}\right)$$

where _*M_t_*_ denotes the mean value of the response variation in the treatment (heterogeneous nutrient supply) and _*M_c_*_ denotes the mean value of the control (homogeneous nutrient supply in dataset 1 and unfertilized treatment in dataset 2).

The approximate variance (*V*_*EZ*_) of each effect size was calculated using eqn 3, and the 100(1-α)% confidence interval (*CI*) of *EZ* was derived using eqn 4:


(3) $$V_{EZ}\approx \frac{{\left(SD\right)}_{t}^{2}}{n_{t}M_{t}^{2}}+\frac{{\left(SD\right)}_{c}^{2}}{n_{c}M_{c}^{2}}$$

(4) $$95\%CI=EZ\pm Z_{\alpha /2}\times \text{SQRT}\left(V_{EZ}\right)$$

where SD is the standard deviation, and _*n_t_*_ and _*n_c_*_ denote the numbers of replicates in, respectively, the treatment and the control on which each *EZ* was based; the value of Z_α/2_ is 1.96 (α = 0.05). If the 95% *CI* values of *EZ* (represented by error bars in the figures) for a given variable did not cover the zero line, the effects of nutrient heterogeneity on the variable were significant. Otherwise, it indicates equal growth of plants or mycorrhizal fungi in heterogeneous nutrient patches and the control.

The effect size from each individual observation was weighted by the reciprocal of *V*_*EZ*_ before being combined in the meta-analysis (Hedges et al., 1999). The overall weighted effects of nutrient supply were derived from the random-effect models (the useful way to estimate the mean effect in a range of studies), considering original estimates as the independent and approximately unbiased samples with known variances (Borenstein et al., 2010). The level of random variation between the heterogeneous nutrient supply effects, known as the residual heterogeneity, was used to estimate whether the categorical factors could explain differences between groups, applying P_*between*_ associated to Q_*between*_ statistics with a mixed-effect model (Viechtbauer, 2007). To test the relationship between the root diameter and foraging precision of roots and mycorrhizal hyphae across the tree species with different mycorrhizal types in experiments with different nutrient patches, we applied linear or second-order polynomial models for each relationship and each group depending on their Akaike information criterion (AIC) values in R version 4.0.5 (R Core Team, 2021).

Publication bias was tested by the funnel plot method (Egger et al., 1997). Even when the existence of publication bias in this meta-analysis was detected, the sensitivity analyses using the trim-and-fill method showed the results were reliable (Duval and Tweedie, 2000).

## Results

Across all the studies reviewed, heterogeneous nutrient supply had a significant positive effect on shoot biomass, root biomass, root foraging precision and mycorrhizal hyphal foraging precision, but a significant negative effect on the effect size on mycorrhizal colonization, and no effect on root:shoot ratio (Table 1). The percentage change of plant biomass allocation (10–12%) was largest in the heterogeneity nutrient treatments compared with homogeneity nutrient control, followed by root foraging precision (6%), while root:shoot ratio was smallest (3%). However, root foraging precision had higher percentage change (14%) than mycorrhizal hyphal foraging precision (6%) and lower than mycorrhizal colonization (24%) in the heterogeneity nutrient treatments compared with unfertilized control.

**TABLE 1:** The statistics for the responses of plant and mycorrhizal fungi traits to nutritionally heterogeneous environments.

**Variables**	**Mean effect size**	**95% CI**	**Percentage change (%)**	**Sample size (n)**
**Dataset 1: heterogeneity nutrient treatments vs. homogeneity nutrient control**				
Shoot biomass	0.111	0.078–0.144	11.74	263
Root biomass	0.096	0.043–0.149	10.08	171
Root:shoot ratio	0.031	−0.024–0.085	3.11	55
Root foraging precision	0.061	0.033–0.089	6.25	180
**Dataset 2: heterogeneity nutrient treatments vs. unfertilized control**				
Root foraging precision	0.134	0.088–0.181	14.39	75
Mycorrhizal hyphal foraging precision	0.055	0.011–0.099	5.63	64
Mycorrhizal colonization	–0.213	−0.329– −0.097	23.70	71

### Shoot and Root Growth

**FIGURE 1:**
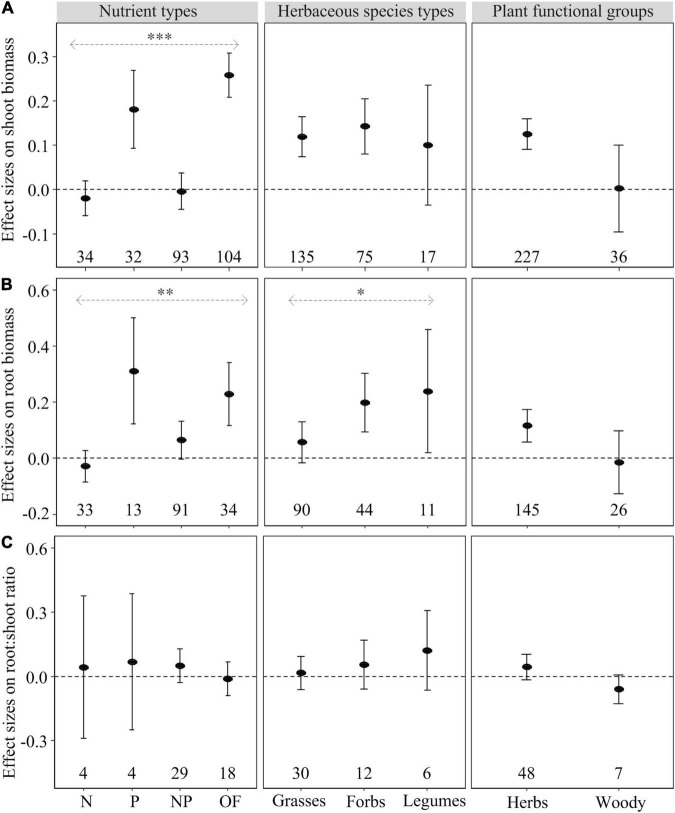
Mean effect sizes (closed circles) of the heterogeneous nutrient supply effects on shoot biomass **(A)**, root biomass **(B)**, and root:shoot ratio **(C)** as influenced by nutrient types (N, P, NP, or OF = organic fertilizer or organic matter), herbaceous species types (grasses, forbs, and legumes), and plant functional groups (herbs and woody) based on the first dataset. Error bars represent ± 95% confidence intervals. Effects are significant if confidence intervals (95% CI) for a given parameter do not overlap with zero. Mean effect sizes < 0 represent a reduction (and >0 an increase) in plant growth in nutritionally heterogeneous environment compared with homogeneous environment. The numbers under the CI bars represent the sample sizes. The asterisks denote significant difference among the categories. ^∗^*P* < 0.05; ^∗∗^*P* < 0.01; ^∗∗∗^*P* < 0.001.

**FIGURE 2:**
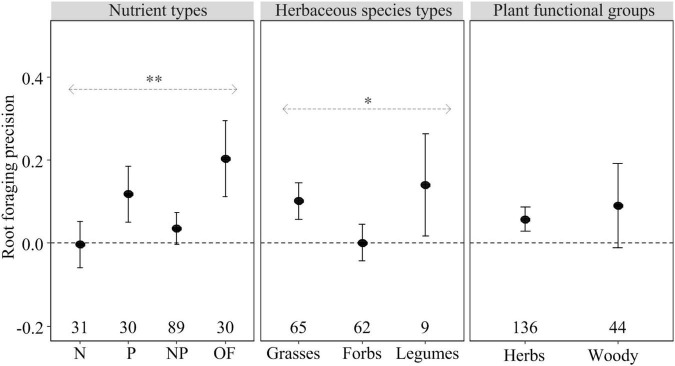
The effect of the heterogeneous nutrient supply on root foraging precision (closed circles) as influenced by nutrient types (N, P, NP, or OF = organic fertilizer or organic matter), herbaceous species types (grasses, forbs, and legumes), and plant functional groups (herbs and woody) based on the first dataset. Error bars represent ± 95% confidence intervals. Effects are significant if confidence intervals (95% CI) for a given parameter do not overlap with zero. Mean effect sizes > 0 represent an increase in plant growth in nutritionally heterogeneous environment compared with homogeneous environment. The numbers under the CI bars represent the sample sizes. The asterisks denote significant difference among the categories. ^∗^*P* ≤ 0.05; ^∗∗^*P* < 0.01.

The effect of heterogeneous nutrient supply on shoot biomass, root biomass and root foraging precision, but not on root:shoot ratio, depended on nutrient types (Figures 1, 2). Heterogeneous P and OF supply significantly increased shoot and root biomass as well as root foraging precision compared to homogeneous nutrient supply (Figures 1, 2). However, heterogeneous N and NP supply had no effect on plant biomass allocation and root foraging precision compared to homogeneous nutrient supply (Figures 1, 2). Regarding root:shoot ratio, no effects of heterogeneously supplied nutrients could be demonstrated (Figure 1C), likely because of relatively small numbers of observations or consistent response trends to nutrients exist in shoots and roots.

Compared with homogeneous nutrient supply, heterogeneous nutrient supply generally increased shoot biomass of grasses and forbs, but not legumes (Figure 1A). In contrast, heterogeneous nutrient supply had a positive effect on root biomass of forbs and legumes, but not grasses (Figure 1B). The root:shoot ratio effects were similar (and non-significant) for grasses, forbs and legumes (Figure 1C). In contrast, Root foraging precision of grasses and legumes rather than forbs was significantly increased in the heterogeneous nutrient treatments (Figure 2).

Heterogeneously supplied nutrients had significant positive effects on shoot and root biomass and root foraging precision of herbaceous species rather than woody species (Figures 1A,B, 2). However, there was no statistically significant difference between the responses of herbaceous and woody species regarding root and shoot biomass, root:shoot ratio and root foraging precision (Figures 1, 2).

### Foraging Traits of Woody Plants

**FIGURE 3:**
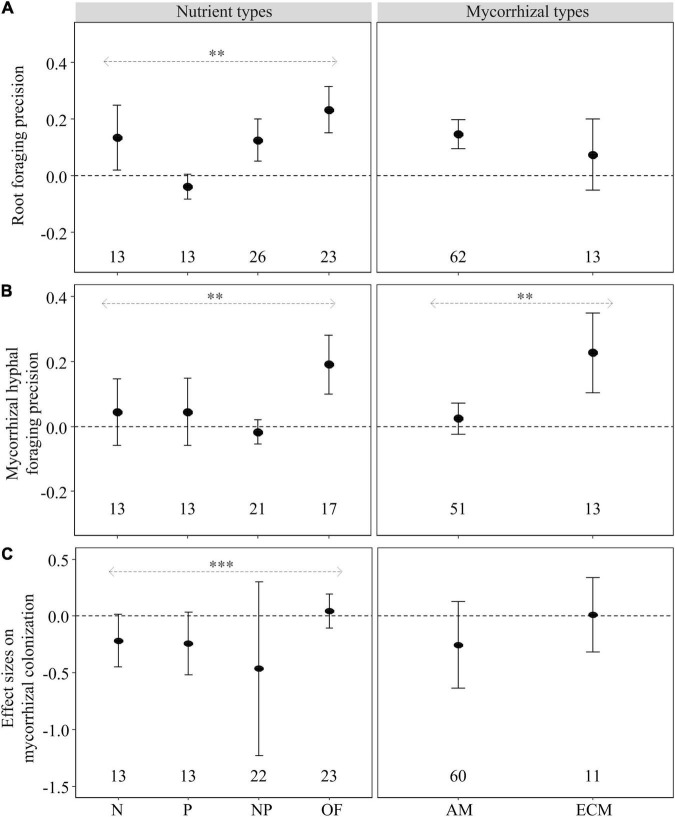
Mean effect sizes (closed circles) of the heterogeneous nutrient supply effects on root proliferation (root foraging precision) **(A)**, mycorrhizal hyphal length or hyphal biomass (mycorrhizal hyphal foraging precision) **(B)** and mycorrhizal colonization **(C)** of woody species as influenced by nutrient types (N, P, NP, or OF = organic fertilizer or organic matter) and mycorrhizal types (AM and ECM) based on the second dataset. Error bars (when larger than the symbol) represent ±95% confidence intervals. Effects are significant if confidence intervals (95% CI) for a given parameter do not overlap with zero. Mean effect sizes < 0 represent a reduction (and >0 an increase) in plant growth in nutritionally heterogeneous patches compared with unfertilized control. The numbers under the CI bars represent the sample sizes. The asterisks denote significant difference among the categories. ^∗∗^*P* < 0.01; ^∗∗∗^*P* < 0.001.

Nutrient type had a significant influence on foraging precision of roots and mycorrhizal hyphae and the effect size of mycorrhizal colonization responding to heterogeneous nutrient patches compared with the unfertilized control (Figure 3). Root foraging precision was significantly increased by heterogeneous N, NP and OF supply, but not by heterogeneous P supply (Figure 3A). Heterogeneous OF supply had significant positive effect on mycorrhizal hyphal foraging precision, but heterogeneous inorganic nutrient supply (i.e., N, P, and NP) had no effect (Figure 3B). No effects of heterogeneously supplied nutrients on the effect size on mycorrhizal colonization could be demonstrated (Figure 3C).

Nutrient heterogeneity increased root foraging precision on AM tree species (Figure 3A) and mycorrhizal hyphal foraging precision of ECM tree species (Figure 3B). Moreover, there was significant difference on mycorrhizal hyphal foraging precision between AM and ECM tree species in the heterogeneity nutrient supply (Figure 3B). However, nutrient heterogeneity had no effect on the effect size on mycorrhizal colonization of AM trees and ECM trees (Figure 3C).

### Correlation of Root Diameter With Foraging Precision of Roots and Mycorrhizal Hyphae

**FIGURE 4:**
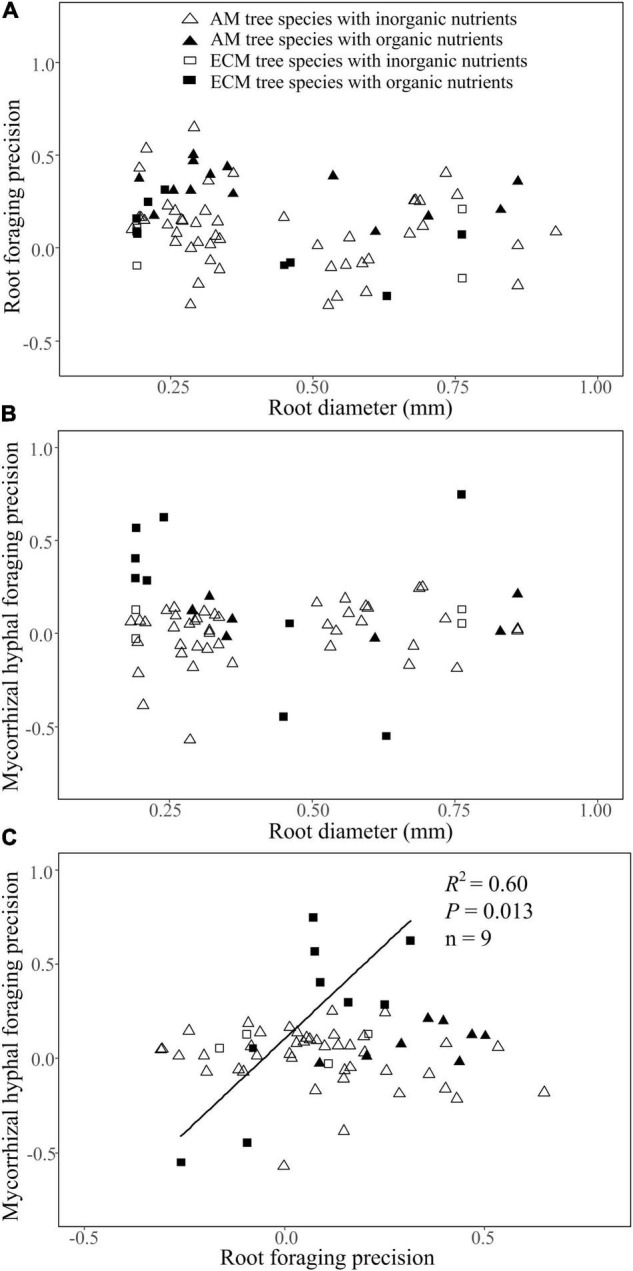
The relationship of root diameter with root foraging precision **(A)** and mycorrhizal hyphal foraging precision **(B)**, and the relationship of root foraging precision with mycorrhizal hyphal foraging precision for AM and ECM woody species **(c)** in organic or inorganic nutrient patches based on the second dataset. Regression lines are shown when *P* < 0.05, and the regression information in **(C)** refers to ECM tree species supplied with organic nutrients heterogeneously (OF).

Root diameter had no correlation with root foraging precision or mycorrhizal hyphal foraging precision of AM or ECM tree species in either inorganic or organic patches (Figures 4A,B). There was a significant positive and linear correlation between root foraging precision and mycorrhizal hyphal foraging precision for ECM trees species in the organic patches rather than inorganic patches, but no clear correlation for AM tree species in either inorganic or organic patches (Figure 4C).

## Discussion

### Effect of Nutrient Types on Plant Responses to Nutrient Heterogeneity

Our meta-analysis showed that nutrient type had large effects on shoot and root biomass in the nutritionally heterogeneous environments (Figure 1). When interpreting the observed response, the context in which the response had been expressed (e.g., attributes of the patch) was as important as the actual response itself (Hodge, 2009). Given the nutrient-specific responses are ubiquitous (e.g., Robinson, 1994a), testing whether plant responses to nutrient patches are phylogenetically and taxonomically conserved across species should be done under the same set of nutrient environments (Kembel and Cahill, 2005).

Our results showed the P and OF patches had relatively strong positive effects on shoot and root biomass and root foraging precision (Figures 1, 2). Because of such nutrient-rich patches persist for relatively long time (Hodge, 2006), root proliferation in the patch zones being critical for enhanced capture immobile nutrients (such as P) and available nutrients released slowly from OF (Hutchings and de Kroon, 1994; Hodge, 2004). Moreover, nutrient immobility in soil also is likely to coincide with a strong plant demand because immobile ions (such as P) have limited transport to the root surface compared with mobile ions (such as NO_3_^–^) (Hodge, 2006). However, the heterogeneous N and NP environment exhibited neutral effects on plant biomass (Figure 1) and root foraging precision (Figure 2). Several factors may be associated with non-response of roots in nutrient-rich patches. Firstly, root response may not be strictly localized in the N-rich patch zone that is mobile (Blouin and Puga-Freitas, 2011). For example, roots of wheat plants did proliferate throughout the whole root system, not just in the patch zone of NO_3_^–^ application (Robinson, 1994b). Secondly, Robinson (1994a) have reviewed “time-dependent responses,” that is, plant responses may occur only after local nutrients have been supplied for a certain time (Dunbabin et al., 2004). Thirdly, the performance of plant roots in the N-rich patches may be related to the local and systemic signaling pathways (Alvarez et al., 2012; Forde, 2014). For mobile ions (such as NO_3_^–^), local resource concentration and root physiological plasticity may be more important than root morphological plasticity (root proliferation) (Dunbabin et al., 2004; Kulmatiski et al., 2017). Finally, Grime and his colleagues also showed that the roots of different species expressed different responses when supplying local nutrients (Grime et al., 1988, 1991), which is consistent with our results (Figures 1B, 2). The roots of some plants failed to respond spectacularly to a nutrient-rich patch may be because they did not have a large demand for that localized nutrient supplied. Hence, the mixed response of plants to heterogeneous nutrient addition is not only related to nutrient type, but also should consider plant functional type.

### Effect of Functional Groups on Plant Responses to Nutrient Heterogeneity

In this meta-analysis, differences in the response patterns between groups of herbaceous species were identified (Figure 1), with shoot biomass of legumes showing no sensitivity to heterogeneous nutrient supply compared to that of grasses and forbs (Figure 1A). This could be associated with legumes symbiotically fixing atmospheric N_2_ to maintain tissue N concentration (Wolf et al., 2017) and having a typically strong capacity to acidify the rhizosphere (soil pH decreasing from 6.5 to 4.1, cf. Li et al., 2007) and increase availability of P in neutral or alkaline soils (Li et al., 2007; Png et al., 2017). These efficient nutrient-capturing strategies in poorly fertile soils may lead to root growth of legumes, rather than shoot biomass, to establish symbiosis with rhizobia (Figures 1B, 2).

For root proliferation, root biomass of grasses was not significantly influenced by heterogeneous nutrient supply (in contrast to forbs and legumes) (Figure 1B). Grass species generally have thinner root diameter and higher specific root length and root mass fraction than other phylogenetic clades, such as forbs (Poorter et al., 2015; Li et al., 2017; Valverde-Barrantes et al., 2017). Hence, proliferation of fine roots in the patch zone may affect mainly root length and have limited influence on the whole root biomass of grasses (Ma et al., 2020). Similarly, Cahill and McNickle (2011) also found grasses place a smaller proportion of root biomass in nutrient patches compared with forbs. In addition, we also found root foraging precision of grass and legume species exhibiting significant positive response to the heterogeneous nutrient supply, but not that of forb species (Figure 2). This indicates that root length has less consistent change with root biomass in heterogeneous nutrient patches.

Compared to herbaceous species, woody plants did not change shoot and root biomass and root foraging precision in nutritionally heterogeneous environments (Figures 1, 2). However, there was no significant difference in effect size between these two functional groups, suggesting their similar biomass allocation and root foraging precision. Conversely, Chen et al. (2018) found much higher root foraging precision in herbaceous than woody plant species. The difference between the two studies may have been caused by the different patch sizes presented to herbs and woody plants. For example, Chen et al. (2018) chose the data of herbaceous species from the study of Johnson and Biondini (2001), whereby the nutrient amount within the patch zone accounted for about 86% of total N and 91% of total P supply, and the nutrient patch as the main nutrient source for plant growth can significantly induce root proliferation (Hodge, 2004). In contrast, the woody species data Chen et al. (2018) chose were mainly from adult trees in a garden (Eissenstat et al., 2015; Chen et al., 2016) and forest (Liu et al., 2015) with small nutrient patch zones (created by root bags or in-growth containers) that can hardly be expected to influence growth of adult trees except for causing local root proliferation only by a local signal from the nutrient-rich zone. As a result, the difference in root plasticity in heterogeneous environments between herbaceous and woody species may have been overestimated in the study by Chen et al. (2018), because of the lack of the integration of the systemic and the local signals in interpreting the root responses to a nutrient patch (see de Kroon et al., 2009). However, nutrient patch sizes we chose in this study were in unison for herbaceous and woody plants, because of 95% of the herb research and all woody plant research in our meta-analysis being conducted in the greenhouse. Thus, explaining the varied root proliferation among plant species remains challenging.

In the present meta-analysis, nutrient supply heterogeneity generally had no influence on root:shoot ratio (Figure 1C and Table 1), regardless of nutrient types and plant functional groups (Figure 1C). Similarly, Wijesinghe et al. (2001) found that only one of six herbaceous species changed root:shoot ratio in the homogeneous vs. heterogeneous environments. This may be due to increased root growth within nutrient patches being generally compensated for by decreased root growth elsewhere (Robinson, 1994a). Moreover, shoot’s rate of growth was slower than root in patch zone and matched that in the uniformly supplied control (Drew and Saker, 1975). Generally, the effects of heterogeneous nutrient supply on root:shoot ratio are complex and difficult to predict from the homogeneous nutrient conditions [see the review by Hutchings and John (2004)]. Overall, the heterogeneous nutrient supply may not influence root:shoot biomass partitioning when the total nutrient amount supplied is the same as in the homogeneous supply conditions.

### Diverse Root and Mycorrhizal Foraging Strategies Among Tree Species

In our meta-analysis, woody plants showed significant root foraging precision with heterogeneous N, NP and OF supply but insignificant with P supply compared with unfertilized control (Figure 3A). In contrast, mycorrhizal hyphal foraging precision exhibited significant positive response to heterogeneous OF supply but non-significant response to heterogeneous inorganic nutrient (such as N, P and NP) supply (Figure 3B). These findings are consistent with many previous studies, which showed that, plants tend to increase root growth rather than mycorrhizal hyphal growth to capture soil available nutrients when nutrients become more freely available (Nilsson and Wallander, 2003; Hodge, 2004; Sharda and Koide, 2010). However, increased proliferation of mycorrhizal hyphae in OF patch may be related to mycorrhizal fungi resisting pathogens through competition for organic nutrients (Zanne et al., 2020).

Our results showed that there was a positive response to nutrient patches by AM tree species regarding root foraging precision and by ECM tree species regarding mycorrhizal hyphae foraging precision (HFP) (Figure 3). Our results were consistent with the study by Chen et al. (2016) who suggested AM tree species relied mostly on root growth and ECM tree species depended mostly on mycorrhizal hyphae to capture nutrients within the nutrient-rich zones. Unexpected, we found that heterogeneous nutrient supply had no effect on the effect size on mycorrhizal colonization for neither among nutrient type nor mycorrhizal type (Figure 3C). Presumably because mycorrhizal colonization is phylogenetic conservative trait (Kong et al., 2014), which is more influenced by host plants than nutrient availability (Šmilauer et al., 2021). Therefore, mycorrhizal hyphal proliferation may be a better indicator than mycorrhizal colonization when determining the effects of mycorrhizal types and heterogeneous nutrient types on mycorrhizal fungi.

In the present study, both root foraging precision and mycorrhizal hyphal foraging precision were independent of root diameter for both AM and ECM tree species in either organic or inorganic patches (Figures 4A,B). This finding suggested that thick-rooted species could have similar root and mycorrhizal hyphal foraging precision as thin-rooted species in a given nutrient type patch. This is consistent with Caplan et al. (2017) who found similar foraging precision among six AM understory shrubs species with differing root diameters and root growth rates. Although the change of root traits has been proposed to influence the root foraging capacity, such as thin root diameter, high specific root length and high root growth rate are associated with high root foraging precision. However, based on our meta-analysis and the published studies, we agree with Hodge (2009) that there may be no simple and definitive “rule” for explaining the variation in root and hyphal proliferation among plant species.

Ectomycorrhizal fungi have the capacity to proliferate hyphae in nutritionally heterogeneous environments (Dowson et al., 1989). However, there was a significant positive linear correlation between mycorrhizal hyphal foraging precision and root foraging precision in organic patches across ECM tree species (Figure 4C). Recent study has demonstrated that ectomycorrhizal mycelial biomass was related to host tree species (Hagenbo et al., 2021). Additionally, compared to AM fungi, ECM fungi have a greater potential for mineralizing organic matter (Smith and Read, 2008; van der Heijden et al., 2015) that may complement the root functions and lead to a close correlation between the root and ECM hyphae foraging strategies. Currently, there is a paucity of data in the literature regarding a relationship between the root and ECM fungi foraging in nutrient patches. The future studies need to focus on clarifying the universality of this foraging pattern in natural forest ecosystems.

### Limitations and Future Research

Recent studies have proposed the interactions between plant species and nutrient types (Liu et al., 2015; Cheng et al., 2016), and between plant and mycorrhizal fungi species (Koch et al., 2017; Hoeksema et al., 2018), influencing the responses of plants and mycorrhizal fungi to nutritionally heterogeneous environments. However, different studies reported a large variation in patch sizes, nutrient partitioning, and mycorrhizal fungi species and genotypes. Even though the present study offered some explanation of adaptive strategies of plant species with different root and mycorrhizae-related traits, our meta-analysis could not completely exclude the influence of the varied experimental factors (such as temperature and precipitation), especially when the available data were limited. Because of a lack of data on the hyphal length or biomass of ECM tree species with large variations in root diameter in inorganic nutrient patches, it remains unclear how root diameter affects foraging strategies of ECM tree species. Hence, there are still large knowledge gaps in predicting plant foraging strategies for different nutrient types from the plant above- and below-ground traits, although our findings partly elucidated some relevant aspects. The future systematic studies should focus on diverse foraging strategies of various plant and mycorrhizal fungi species, taking into account evolution perspectives, in order to provide deep understanding of the relationships governing plant species coexistence.

## Conclusion

By synthesizing 879 observations from 66 studies, our meta-analysis results showed that nutrient types explained most of the variation in the plant and mycorrhizal fungi responses to nutritionally heterogeneous environments. No significant difference was noted in responses to nutrient heterogeneity among functional groups of herbs (grasses, forbs, and legumes), between herbs and woody plants, and between AM and ECM tree species regarding plant growth and foraging strategy, except for root biomass and root foraging precision among grasses, forbs and legumes, and mycorrhizal hyphal foraging precision between AM and ECM tree species. Root foraging precision and mycorrhizal hyphal foraging precision had no correlation with root diameter, regardless of the nutrient type and mycorrhizal type. In addition, root foraging precision was positively correlated with mycorrhizal hyphal foraging precision for ECM tree species in the organic patches. The results of our meta-analysis suggest that nutrient type mainly regulate plant response to heterogeneous nutrient supplies, although AM tree species enhanced root foraging precision and ECM tree species improved mycorrhizal hyphal foraging precision.

## Data Availability Statement

All data generated or analyzed during this study are included in the article and Supplementary Material.

## Author Contributions

BL, FH, KX, and AZ conceived the ideas and collected and analyzed the data. All authors interpreted the data and revised the manuscript.

## Conflict of Interest

The authors declare that the research was conducted in the absence of any commercial or financial relationships that could be construed as a potential conflict of interest.

## Publisher’s Note

All claims expressed in this article are solely those of the authors and do not necessarily represent those of their affiliated organizations, or those of the publisher, the editors and the reviewers. Any product that may be evaluated in this article, or claim that may be made by its manufacturer, is not guaranteed or endorsed by the publisher.

## Footnotes

1
http://getdata-graph-digitizer.com

## Supplementary Material

Supplementary Appendix 1 Dataset (excel file) on plant biomass allocation and root foraging strategy in the first dataset (heterogeneous nutrient vs. homogeneous nutrient control), and foraging strategy of roots and mycorrhizal fungi and their relationship with root diameter for woody species in the second dataset (heterogeneous nutrient patch vs. unfertilized control). AM: arbuscular mycorrhizal fungi; ECM: ectomycorrhizal fungi.

Supplementary Appendix 2 The articles from which data selected in this meta-analysis (text document).
