# Supplementary material for: The Response of Plants and Mycorrhizal Fungi to Nutritionally-Heterogeneous Environments Are Regulated by Nutrient Types and Plant Functional Groups
Source: Front Plant Sci. 2021 Nov 15;12:734641. doi: 10.3389/fpls.2021.734641 (PMC8634332; doi:10.3389/fpls.2021.734641)
Supplement: Supplementary Appendix 2 — The articles from which data selected in this meta-analysis (text document). [file Table_2.doc]

**Full listing of 66 publications (61 for the first dataset and 5 for the second dataset) included in the meta-analysis.**

**References**

Blair, B. (2001). Effect of soil nutrient heterogeneity on the symmetry of belowground competition. *Plant Ecol.* 156, 199–203. doi:10.1023/A:1012664431933.

Blair, B. C., and Perfecto, I. (2004). Successional status and root foraging for phosphorus in seven tropical tree species. *Can. J. For. Res.* 34, 1128–1135. doi:10.1139/x04-004.

Bliss, K. M., Jones, R. H., Mitchell, R. J., and Mou, P. P. (2002). Are competitive interactions influenced by spatial nutrient heterogeneity and root foraging behavior? *New Phytol.* 154, 409–417. doi:10.1046/j.1469-8137.2002.00389.x.

Blouin, M., and Puga-Freitas, R. (2011). Combined effects of contrast between poor and rich patches and overall nitrate concentration on Arabidopsis thaliana root system structure. *Funct. Plant Biol.* 38, 364–371. doi:10.1071/FP10232.

Caplan, J. S., Stone, B. W. G., Faillace, C. A., Lafond, J. J., Baumgarten, J. M., Mozdzer, T. J., et al. (2017). Nutrient foraging strategies are associated with productivity and population growth in forest shrubs. *Ann. Bot.* 119, 977–988. doi:10.1093/aob/mcw271.

Casper, B. B., and Cahill Jr, J. F. (1998). Population‐level responses to nutrient heterogeneity and density by *Abutilon theophrasti* (Malvaceae): an experimental neighborhood approach. *Am. J. Bot.* 85, 1680–1687.

Chen, W., Koide, R. T., Adams, T. S., DeForest, J. L., Cheng, L., and Eissenstat, D. M. (2016). Root morphology and mycorrhizal symbioses together shape nutrient foraging strategies of temperate trees. *Proc. Natl. Acad. Sci. U. S. A.* 113, 8741–8746. doi:10.1073/pnas.1601006113.

Cheng, L., Chen, W., Adams, T. S., Wei, X., Li, L., Luke, M., et al. (2016). Mycorrhizal fungi and roots are complementary in foraging within nutrient patches. *Ecology* 97, 2815–2823.

Crabtree, R. C., and Bazzaz, F. A. (1992). Seedlings of black birch (*Betula lenta* L.) as foragers for nitrogen. *New Phytol.* 122, 617–625. doi:10.1111/j.1469-8137.1992.tb00089.x.

Cui, M., and Caldwell, M. M. (1996a). Facilitation of plant phosphate acquisition by arbuscular mycorrhizas from enriched soil patches I. Roots and hyphae exploiting the same soil volume. *New Phytol.* 133, 461–467. doi:10.1111/j.1469-8137.1996.tb01913.x.

Cui, M., and Caldwell, M. M. (1996b). Facilitation of plant phosphate acquisition by arbuscular mycorrhizas from enriched soil patches II. Hyphae exploiting root-free soil. *New Phytol.* 133, 461–467. doi:10.1111/j.1469-8137.1996.tb01913.x.

Cui, M., and Caldwell, M. M. (1997). Shading reduces exploitation of soil nitrate and phosphate by *Agropyron desertorum* and *Artemisia tridentata* from soils with patchy and uniform nutrient distributions. *Oecologia* 109, 177–183. doi:10.1007/s004420050072.

Cui, M., and Caldwell, M. M. (1998). Nitrate and phosphate uptake by *Agropyron desertorum* and *Artemisia tridentata* from soil patches with balanced and unbalanced nitrate and phosphate supply. *New Phytol.* 139, 267–272. doi:10.1046/j.1469-8137.1998.00188.x.

Day, K. J., Hutchings, M. J., and John, E. A. (2003a). The effects of spatial pattern of nutrient supply on yield, structure and mortality in plant populations. *J. Ecol.* 91, 541–553. doi:10.1046/j.1365-2745.2003.00799.x.

Day, K. J., John, E. A., and Hutchings, M. J. (2003b). The effects of spatially heterogeneous nutrient supply on yield, intensity of competition and root placement patterns in *Briza media* and *Festuca ovina*. *Funct. Ecol.* 17, 454–463. doi:10.1046/j.1365-2435.2003.00758.x.

Dong, B.-C., Wang, J.-Z., Liu, R.-H., Zhang, M.-X., Luo, F.-L., and Yu, F.-H. (2015). Soil heterogeneity affects ramet placement of Hydrocotyle vulgaris. *J. Plant Ecol.* 8, 91–100. doi:10.1093/jpe/rtu003.

Einsmann, J. C., Jones, R. H., Pu, M., and Mitchell, R. J. (1999). Nutrient foraging traits in 10 co-occurring plant species of contrasting life forms. *J. Ecol.* 87, 609–619. doi:10.1046/j.1365-2745.1999.00376.x.

Eissenstat, D. M., Kucharski, J. M., Zadworny, M., Adams, T. S., and Koide, R. T. (2015). Linking root traits to nutrient foraging in arbuscular mycorrhizal trees in a temperate forest. *New Phytol.* 208, 114–124. doi:10.1111/nph.13451.

Facelli, E., and Facelli, J. M. (2002). Soil phosphorus heterogeneity and mycorrhizal symbiosis regulate plant intra-specific competition and size distribution. *Oecologia* 133, 54–61. doi:10.1007/s00442-002-1022-5.

Felderer, B., Boldt-Burisch, K. M., Schneider, B. U., Hüttl, R. F. J., and Schulin, R. (2013). Root growth of *Lotus corniculatus* interacts with P distribution in young sandy soil. *Biogeosciences* 10, 1737–1749. doi:10.5194/bg-10-1737-2013.

Fransen, B., Blijjenberg, J., and De Kroon, H. (1999). Root morphological and physiological plasticity of perennial grass species and the exploitation of spatial and temporal heterogeneous nutrient patches. *Plant Soil* 211, 179–189. doi:10.1023/A:1004684701993.

Fransen, B., De Kroon, H., and Berendse, F. (2001). Soil nutrient heterogeneity alters competition between two perennial grass species. *Ecology* 82, 2534–2546. doi:10.1890/0012-9658(2001)082[2534:SNHACB]2.0.CO;2.

García-Palacios, P., Maestre, F. T., and Gallardo, A. (2011). Soil nutrient heterogeneity modulates ecosystem responses to changes in the identity and richness of plant functional groups. *J. Ecol.* 99, 551–562. doi:10.1111/j.1365-2745.2010.01765.x.

Gloser, V., Libera, K., and Orians, C. M. (2008). Contrasting below- and aboveground responses of two deciduous trees to patchy nitrate availability. *Tree Physiol.* 28, 37–44. doi:10.1093/treephys/28.1.37.

Hagiwara, Y., Kachi, N., and Suzuki, J. I. (2012). Effects of temporal heterogeneity of water supply and nutrient levels on plant biomass growth depend on the plant’s relative size within its population. *Ecol. Res.* 27, 1079–1086. doi:10.1007/s11284-012-0989-6.

He, W. M., Shen, Y., and Cornelissen, H. H. C. (2012). Soil nutrient patchiness and plant genotypes interact on the production potential and decomposition of root and shoot litter: Evidence from short-term laboratory experiments with *Triticum aestivum*. *Plant Soil* 353, 145–154. doi:10.1007/s11104-011-1018-1.

He, Y., Liao, H., and Yan, X. (2003). Localized supply of phosphorus induces root morphological and architectural changes of rice in split and stratified soil cultures. *Plant Soil* 248, 247–256. doi:10.1023/A:1022351203545.

Hodge, A., Stewart, J., Robinson, D., Griffiths, B. S., and Fitter, A. H. (1998). Root proliferation, soil fauna and plant nitrogen capture from nutrient-rich patches in soil. *New Phytol.* 139, 479–494. doi:10.1046/j.1469-8137.1998.00216.x.

Hou, X., Tigabu, M., Zhang, Y., Ma, X., Cai, L., Wu, P., et al. (2017). Root plasticity, whole plant biomass, and nutrient accumulation of Neyraudia reynaudiana in response to heterogeneous phosphorus supply. *J. Soils Sediments* 17, 172–180. doi:10.1007/s11368-016-1517-z.

Hutchings, M. J., and Wijesinghe, D. K. (2008). Performance of a clonal species in patchy environments: Effects of environmental context on yield at local and whole-plant scales. *Evol. Ecol.* 22, 313–324. doi:10.1007/s10682-007-9178-4.

Jackson, R. B., Manwaring, J. H., and Caldwell, M. M. (1990). Rapid physiological adjustment of roots to localized soil enrichment. *Nature* 344, 58–60.

James, J. J., Mangold, J. M., Sheley, R. L., and Svejcar, T. (2009). Root plasticity of native and invasive Great Basin species in response to soil nitrogen heterogeneity. *Plant Ecol.* 202, 211–220. doi:10.1007/s11258-008-9457-3.

Jansen, C., Van De Steeg, H. M., and De Kroon, H. (2005). Investigating a trade-off in root morphological responses to a heterogeneous nutrient supply and to flooding. *Funct. Ecol.* 19, 952–960. doi:10.1111/j.1365-2435.2005.01049.x.

Jansen, C., Van Kempen, M. M. L., Bögemann, G. M., Bouma, T. J., and De Kroon, H. (2006). Limited costs of wrong root placement in *Rumex palustris* in heterogeneous soils. *New Phytol.* 171, 117–126. doi:10.1111/j.1469-8137.2006.01733.x.

Jing, J., Rui, Y., Zhang, F., Rengel, Z., and Shen, J. (2010). Localized application of phosphorus and ammonium improves growth of maize seedlings by stimulating root proliferation and rhizosphere acidification. *F. Crop. Res.* 119, 355–364. doi:10.1016/j.fcr.2010.08.005.

Lamb, E. G., Haag, J. J., and Cahill, J. F. (2004). Patch-background contrast and patch density have limited effects on root proliferation and plant performance in *Abutilon theophrasti*. *Funct. Ecol.* 18, 836–843. doi:10.1111/j.0269-8463.2004.00893.x.

Lamb, E. G., Stewart, A. C., and Cahill, J. F. (2012). Root system size determines plant performance following short-term soil nutrient pulses. *Plant Ecol.* 213, 1803–1812. doi:10.1007/s11258-012-0135-0.

Li, H., Ma, Q., Li, H., Zhang, F., Rengel, Z., and Shen, J. (2014). Root morphological responses to localized nutrient supply differ among crop species with contrasting root traits. *Plant Soil* 376, 151–163. doi:10.1007/s11104-013-1965-9.

Liu, B., Li, L., Rengel, Z., Tian, J., Li, H., and Lu, M. (2019). Roots and arbuscular mycorrhizal fungi are independent in nutrient foraging across subtropical tree species. *Plant Soil* 442, 97–112. doi:10.1007/s11104-019-04161-3.

Loecke, T. D., and Robertson, G. P. (2009). Soil resource heterogeneity in the form of aggregated litter alters maize productivity. *Plant Soil* 325, 231–241. doi:10.1007/s11104-009-9973-5.

Ma, Q., Rengel, Z., and Siddique, K. H. M. (2011). Wheat and white lupin differ in root proliferation and phosphorus use efficiency under heterogeneous soil P supply. *Crop Pasture Sci.* 62, 467–473. doi:10.1071/CP10386.

Mackie-Dawson, L. A. (1999). Nitrogen uptake and root morphological responses of defoliated *Lolium perenne* (L.) to a heterogeneous nitrogen supply. *Plant Soil* 209, 111–118. doi:10.1023/A:1004534609280.

Maestre, F. T., Bradford, M. A., and Reynolds, J. F. (2006). Soil heterogeneity and community composition jointly influence grassland biomass. *J. Veg. Sci.* 17, 261-270. doi:10.1658/1100-9233(2006)017[0261:shaccj]2.0.co;2.

Maestre, F. T., Quero, J. L., Valladares, F., and Reynolds, J. F. (2007). Individual vs. population plastic responses to elevated CO2, nutrient availability, and heterogeneity: A microcosm experiment with co-occurring species. *Plant Soil* 296, 53–64. doi:10.1007/s11104-007-9289-2.

Maestre, F. T., and Reynolds, J. F. (2006a). Small-scale spatial heterogeneity in the vertical distribution of soil nutrients has limited effects on the growth and development of *Prosopis glandulosa* seedlings. *Plant Ecol.* 183, 65–75. doi:10.1007/s11258-005-9007-1.

Maestre, F. T., and Reynolds, J. F. (2006b). Nutrient availability and atmospheric CO2 partial pressure modulate the effects of nutrient heterogeneity on the size structure of populations in grassland species. *Ann. Bot.* 98, 227–235. doi:10.1093/aob/mcl093.

Maestre, F. T., and Reynolds, J. F. (2007). Biomass responses to elevated CO_2_, soil heterogeneity and diversity: An experimental assessment with grassland assemblages. *Oecologia* 151, 512–520. doi:10.1007/s00442-006-0577-y.

Mou, P., Jones, R. H., Tan, Z., Bao, Z., and Chen, H. (2013). Morphological and physiological plasticity of plant roots when nutrients are both spatially and temporally heterogeneous. *Plant Soil* 364, 373–384. doi:10.1007/s11104-012-1336-y.

Mou, P., Mitchell, R. J., and Jones, R. H. (1997). Root distribution of two tree species under a heterogeneous nutrient environment. *J. Appl. Ecol.* 34, 645. doi:10.2307/2404913.

Nakamura, R., Kachi, N., and Suzuki, J. I. (2008). Root growth and plant biomass in *Lolium perenne* exploring a nutrient-rich patch in soil. *J. Plant Res.* 121, 547–557. doi:10.1007/s10265-008-0183-7.

Neatrour, M. A., Jones, R. H., and Golladay, S. W. (2007). Response of three floodplain tree species to spatial heterogeneity in soil oxygen and nutrients. *J. Ecol.* 95, 1274–1283. doi:10.1111/j.1365-2745.2007.01304.x.

Rajaniemi, T. K., and Reynolds, H. L. (2004). Root foraging for patchy resources in eight herbaceous plant species. *Oecologia* 141, 519–525. doi:10.1007/s00442-004-1666-4.

Rose, T. J., Rengel, Z., Ma, Q., and Bowden, J. W. (2009). Crop species differ in root plasticity response to localised P supply. *J. Plant Nutr. Soil Sci.* 172, 360–368. doi:10.1002/jpln.200800031.

Schortemeyer, M., and Feil, B. (1996). Root morphology of maize under homogeneous or spatially separated supply of ammonium and nitrate at three concentration ratios. *J. Plant Nutr.* 19, 1089–1097. doi:10.1080/01904169609365182.

Si, C., Xue, W., Guo, Z. W., Zhang, J. F., Hong, M. M., Wang, Y. Y., et al. (2021). Soil heterogeneity and earthworms independently promote growth of two bamboo species. *Ecol. Indic.* 130, 108068. doi:10.1016/j.ecolind.2021.108068.

Stevens, G. N., and Jones, R. H. (2006). Influence of root herbivory on plant communities in heterogeneous nutrient environments. *New Phytol.* 171, 127–136. doi:10.1111/j.1469-8137.2006.01731.x.

Trapeznikov, V. K., Ivanov, I. I., and Kudoyarova, G. R. (2003). Effect of heterogeneous distribution of nutrients on root growth, ABA content and drought resistance of wheat plants. *Plant Soil* 252, 207–214. doi:10.1023/A:1024734310214.

van Vuuren, M. M. I., Muir, A. A., and Orians, C. M. (2003). Growth and nutrient uptake by birch and maple seedlings on soil with patchy or homogeneous distributionof organic matter. *Can. J. For. Res.* 33, 2019–2026. doi:10.1139/X03-128

Visser, E. J. W., Bögemann, G. M., Smeets, M., De Bruin, S., De Kroon, H., and Bouma, T. J. (2008). Evidence that ethylene signalling is not involved in selective root placement by tobacco plants in response to nutrient-rich soil patches. *New Phytol.* 177, 457–465. doi:10.1111/j.1469-8137.2007.02256.x.

Wang, L., De Kroon, H., and Smits, A. J. M. (2007). Combined effects of partial root drying and patchy fertilizer placement on nutrient acquisition and growth of oilseed rape. *Plant Soil* 295, 207–216. doi:10.1007/s11104-007-9276-7.

Wang, P., Hasnain, M., Cahill, J., and Wu, D. (2020). The multi-response of root foraging strategy to a neighbor, soil heterogeneity and earthworm. *Appl. Soil Ecol.* 155, 103684. doi:10.1016/j.apsoil.2020.103684.

Wijesinghe, D. K., John, E. A., Beurskens, S., and Hutchings, M. J. (2001). Root system size and precision in nutrient foraging: Responses to spatial pattern of nutrient supply in six herbaceous species. *J. Ecol.* 89, 972–983. doi:10.1046/j.0022-0477.2001.00618.x.

Wu, B., Fullen, M. A., Li, J., An, T., Fan, Z., Zhou, F., et al. (2014). Integrated response of intercropped maize and potatoes to heterogeneous nutrients and crop neighbours. *Plant Soil* 374, 185–196. doi:10.1007/s11104-013-1865-z.

Zhang, D., Zhang, C., Tang, X., Li, H., Zhang, F., Rengel, Z., et al. (2016). Increased soil phosphorus availability induced by faba bean root exudation stimulates root growth and phosphorus uptake in neighbouring maize. *New Phytol.* 209, 823–831. doi:10.1111/nph.13613.

Zhang, Y., Zhou, Z., Ma, X., and Jin, G. (2010). Foraging ability and growth performance of four subtropical tree species in response to heterogeneous nutrient environments. *J. For. Res.* 15, 91–98. doi:10.1007/s10310-009-0153-5.

Zhou, J., Dong, B. C., Alpert, P., Li, H. L., Zhang, M. X., Lei, G. C., et al. (2012). Effects of soil nutrient heterogeneity on intraspecific competition in the invasive, clonal plant *Alternanthera philoxeroides*. *Ann. Bot.* 109, 813–818. doi:10.1093/aob/mcr314.
